# An Approach to a Silver Conductive Ink for Inkjet Printer Technology

**DOI:** 10.3390/polym16121731

**Published:** 2024-06-18

**Authors:** Svetlana N. Kholuiskaya, Valentina Siracusa, Gulnaz M. Mukhametova, Luybov A. Wasserman, Vladislav V. Kovalenko, Alexey L. Iordanskii

**Affiliations:** 1 N.N. Semenov Federal Research Center for Chemical Physics, Russian Academy of Science (RAS), 4 Kosygina St., 119991 Moscow, Russia; mukhametova@mail.ru (G.M.M.); vladislavkovalenko785@gmail.com (V.V.K.); aljordan08@gmail.com (A.L.I.); 2 Department of Chemical Science (DSC), University of Catania, Viale A. Doria 6, 95125 Catania, Italy; 3 Emanuel Institute of Biochemical Physics, RAS, 4 Kosygina St., 119334 Moscow, Russia; lwasserma@mail.ru

**Keywords:** reactive silver ink, inkjet printing, high conductivity, silver layer

## Abstract

Silver-based metal–organic decomposition inks composed of silver salts, complexing agents and volatile solvents are now the subject of much research due to the simplicity and variability of their preparation, their high stability and their relatively low sintering temperature. The use of this type of ink in inkjet printing allows for improved cost-effective and environmentally friendly technology for the production of electrical devices, including flexible electronics. An approach to producing a silver salt-based reactive ink for jet printing has been developed. The test images were printed with an inkjet printer onto polyimide substrates, and two-stage thermal sintering was carried out at temperatures of 60 °C and 100–180 °C. The structure and electrical properties of the obtained conductive lines were investigated. As a result, under optimal conditions an electrically conductive film with low surface resistance of approximately 3 Ω/square can be formed.

## 1. Introduction

As a low-cost technology, inkjet printing provides a novel approach for the fabrication of functional printable materials in electronics [1], innovative platforms for drug delivery [2], high-throughput DNA synthesis [3], flexible sensors and electronic devices [4,5], QR code design [6] and in many other polymer industries. This printing technology borrows principles from the graphic industry to produce conductive patterns on any surface controlled by computer programs. Important advantages of this technology are both the miniaturization and deposition of ink on large areas, as well as the flexibility of printable products. It is important that the substrates for printing are varied in a wide range including polymers, paper, textiles, biomaterials, concretes, etc. [7,8,9]. Flexible printed electronics are suitable for designing electrodes, displays and antenna arrays, sensors, batteries, photovoltaic cells, radio frequency identification (RFID) tags, light-emitting diodes (LEDs) [10,11,12,13,14,15,16,17,18,19], biomedical devices for deep-tissue sensors [20] and in electronic skins [21], and could be used in the future for biomedical devices.

It is worth noting that the post-treatment of printed circuit boards is a necessary step to transform them into conductive ones. Thermal sintering is often used and usually carried out at moderate to high temperatures, 90–250 °C, during the time interval required to evaporate organic solvents and to decompose harmful additives. Due to the temperature sensitivity of the most suitable substrates, alternative, low-temperature non-contact photonic technologies such as photonic sintering, plasma sintering and microwave sintering have been intensively studied [22,23,24,25]. However, these approaches still have their own disadvantages, such as expensive equipment, complicated procedures and more. Many printable devices are too sophisticated for large-scale preparation in industry. Therefore, thermal sintering remains the most common and cheapest post-treatment method. Obviously, the key component of printing is conductive ink. Owing to the development of nanoscale inorganic substances, the range of functional materials for conductive inks has greatly expanded. It includes classes such as graphene and its derivatives, carbon nanotubes, conducting polymers, nanoparticles and metal nanowires, as well as their complexes [26,27,28,29,30,31,32,33]. Inkjet printing inks must have a high degree of control over material properties (e.g., viscosity, surface tension, adhesion to various surfaces) and especially electrical conductivity as a principal characteristic. Due to their excellent electrical properties, metals, namely copper and silver, are considered the most promising materials. Copper is inexpensive but oxidizes easily in contact with air, making pure metal difficult to obtain and increasing resistance of the printed pattern [34,35]. Additionally, copper salts used for inks are thermostable and decompose at a relatively high temperature, around 200 °C, and cannot be used with polymers and other flexible materials for printing via the thermal sintering technique [33]. Moreover, the mass fraction of copper in the precursors is low, which necessitates repeated patterning. After comparison with copper and a number of other metals, a silver-based ink formulation with appropriate electrical conductivity and stable chemical properties should be considered as a suitable candidate for inkjet printing [36,37].

Two principal approaches to silver ink formulation have been developed. In the first case, inks consist of highly concentrated dispersions of silver nanoparticles or nanowires. Typically, volatile solvents and various additives are also used to adjust the viscosity and contact angle for print optimization. Moreover, stabilization with various additives, which are often organic polymers, is necessary to prevent particle aggregation that occurs during long-term storage. As a result, nanosilver inks have a high annealing temperature (>200 °C) that allows experts to remove stabilizing agents. Harsh annealing conditions narrow the choice of substrate. Another limitation to their wide application relates to the particle size and poorly reproducible performance of tracks. During printing, particle-based inks could easily cause clogging of nozzles in the print head [38]; therefore, it is reasonable to use particles with a diameter of 50 nm or less [39,40]. Nevertheless, even particles of this size easily agglomerate in the synthesis, printing and storage processes. To eliminate the above problems, various approaches are being developed. A recent development is the creation of hybrid technologies, combining, for example, nanosilver particles with graphene [41]. The high conductivity of silver, together with the excellent mechanical properties of graphene, results in a low-silver ink that is equal in electrical properties to the best examples of silver ink [42]. Another work [43] presents the original idea of ink using AgNPs and a water-based ultraviolet curable resin. The proposed composition allows for obtaining AgNPs of optimal size and conducting UV curing and sintering at low temperatures, which offers the prospect of using this ink for flexible electronics. It is also worth noting the promising work [44] describing 3D printing ink formulated as an emulsion of environmentally friendly components: bee pollen microparticles, citric acids and CuNPs. Printed patterns dried under ambient conditions represent an organic framework with the inclusion of a small number (<10 vol%) of copper particles. Samples of this size have excellent electrical conductivity, as well as very low density. The combination of these advantages makes the formulated ink very useful for the production of electrical devices using 3D printing. Although successful examples have been presented for overcoming AgNP-based ink issues, the most widely accepted approach is to use metal–organic decomposition (MOD) inks, also called reactive inks.

Silver ions in MOD ink are stabilized in a soluble complex with specific ligands that can be easily removed at mild temperatures. These solution-phase inks are free of particle agglomeration since the metal exists in ionic form. The thermal decomposition process includes reduction of the central metal ion, normally with the salt anion, coordinating groups or some reducing agents. As a result, a conductive layer appears during nucleation and grows with or without nanoparticles. For the formulation of reactive inks, silver precursors are required in the form of metal complexes with high solubility and low decomposition temperature. Considering these requirements, carboxylates and their derivatives are preferred as complexing agents. They can not only be used as starting reagents, but can also be formed in situ. Some initial silver compounds, namely nitrates, carbonates, oxides and others, have been tested too. Early studies examined silver neodecanoate [45,46], systems of silver nitrate–1-dimethylamino-2-propanol [47], silver oxide–ammonium carbomate [48] and silver oxide–diethanolamin [49]. All of these MOD-based inks provided satisfactory conductivity, but sintering temperatures were still relatively high, above 100 °C. Later, many researchers’ efforts were inspired by Walker et al. [50], who produced a reactive silver ink by the modified Tollen process in which the main active agent is the precursor Ag(NH_3_)_2_CH_3_CO_2_. The inks were produced from silver acetate and contained 22 wt.% Ag. This technique exhibited a remarkable result: the conductivity of printed lines after annealing at 90 °C was close to that of bulk silver. The central metal ion of the precursor in this MOD-based ink is stabilized by amino alcohol. Developing this approach, various amino alcohols with silver acetate [51,52,53,54], silver carbonate [55] and silver oxide [55] as initial compounds were used. Ammonia and various types of amines have also been introduced as complexing agents [56,57,58,59]. Silver ions in the ink were reduced in some cases by formic acid [51,52,56], aldehydes [49,60], hydroquinone [46] and glycol [55]. Even low-molecular-weight alcohols, which are usually selected as solvents due to their easy evaporation, have reduction capability. Several additives have proven to be effective; for example, hydroxyl ethyl cellulose (HEC) [52] have been used to improve the solution properties of MOD-based inks.

Overall, some progress has been made in developing inks with desired properties, especially at low sintering temperatures. Thus, it was possible to reduce them to 75 °C [49] and even to room temperature [53]. However, none of the works achieved the closed conductivity of bulk silver, as in the method of Walker et al. This is due, among other things, to the complication of multi-component ink composition, which led to a decrease in the mass fraction of silver.

Walker et al. fabricated conductive silver patterns by printing reactive ink on different polymeric substrates, glass and cellulose-based materials [51]. Subsequently, the range of substrates and sintering techniques was expanded. Conductive silver patterns have been prepared on textiles [61], on polyimide (PI) by a laser direct writing technique [24], on paper by atmospheric pressure plasma sintering [62] and on PI using a combination of thermal and plasma sintering [63]. The work reported above demonstrates the wide range of applications of Walker’s inks, with variable results depending on sintering conditions. Under thermal processing, the electrical conductivity of printed silver films has been observed to be enhanced with increased sintering temperature [52,60,64,65]. However, the influence of temperature is not always so unambiguous. Xie et al. [54] obtained an interesting result by studying reactive inks based on amino alcohols with different numbers of hydroxyl groups. The boiling point of amino alcohols directly depends on the number of hydroxyl groups. The authors demonstrated that amino alcohols with two and three hydroxyl groups, due to slower evaporation, form more uniform silver domains on the surface than ethanolamine, and this results in better conductivity. However, the use of diethanolamine is preferable because it evaporates from the surface more easily than triethanolamine.

Our work is aimed at developing functional silver inks applied in inkjet technology. This study provides the synthetic route of a silver-based metal–organic decomposition ink and adapts it for printing on flexible polymeric substrates, such as polyimide film. The authors prepared a stable reactive silver ink based on the modified Tollen technique formulation, in which ammonia and formic acid act as ligands of the silver acetate precursor. Highly conductive printed silver patterns on polyimide substrate with a chemically modified surface were fabricated after two-step annealing. The conductive lines showed good adhesion and stability. We investigated the influence of printing steps and sintering temperature on the electrical resistivity and morphology of inkjet-printed patterns. We found that low-temperature pre-baking was necessary to achieve a smooth surface which resulted in low resistivity of silver circuits. Additionally, we examined ink solutions for their content of colloidal silver particles as possible seeds of metallic silver crystallization during thermal processing.

## 2. Materials and Methods

### 2.1. Materials

All chemicals—silver acetate (ASC reagent grade, anhydrous, 98% purity), ammonium hydroxide (reagent grade, 28–30 wt%), formic acid (ASC reagent grade, ≥96%) and sodium hydroxide (reagent grade, powder, 97%)—were obtained from Sigma-Aldrich Company, Darmstadt, Germany (the supplier Merk Life Science LLC, Saint Petersburg, Russia) and used without further purification. Polyimide film was purchased from Kehu Electric (Sverdlovsk Oblast, Russia).

### 2.2. Conductive Ink Preparation

To prepare the ink, a 1.0 g silver acetate suspension was dissolved in 4.0 mL of aqueous ammonia, added dropwise during vigorous stirring, and cooled in a water bath at t = 5 °C. After the dissolution of silver acetate, 0.15 mL of formic acid was also added to the solution under stirring, without heating, and the solution immediately turned dark gray. Stirring was continued for 10 min, then the solution was stored at 22 °C for two hours and filtered through a material with a 0.45-micron pore size. The resulting solution was stored in the refrigerator at t = 3 ÷ 5 °C. The next day, no sediment was released and the ink acquired a transparent color, probably due to the equilibrium processes of suspension ordering. The ink was filtered again before each application. The amount of silver mass in the ink was determined gravimetrically. Silver mass concentration was determined after heat treating the ink sample (0.243 g) at 150 °C for 30 min and weighing the silver sediment, which was 0.034 g. Ink viscosity was measured on a Brookfield NDJ-8S viscometer (Shenzhen Graigar Technology Co., Shenzhen, China); surface tension was determined using a ZWZL300 Interfacial Tension Tester (Baoding Zhiwei Electric Power Technology Co., Baoding, China).

### 2.3. Physico-Chemical Methods in the Research and Printing Processes

Drying and sintering of the ink on a polyimide film was carried out using a Primlab PL-R-steps-H (RF) magnetic stirrer equipped with a closed thermostatically controlled aluminum unit with a temperature control accuracy of 0.1 °C. The typical sintering time was 30 min for all samples. To increase the polyimide film surface hydrophilicity, it was kept in a 5% NaOH solution for 2 h, washed with distilled water and acetone and subsequently air-dried.

The 3D printer Voltera V-One (Waterloo, ON, Canada) was used for printing.

The surface resistivity of the samples was measured by a 4-probe method on a milliohmmeter ST-2258C (Suzhou Jingge Electronics, Suzhou, China) after sintering. Each sample was measured in 10 runs. The average value was determined for each sample.

The morphology and thickness of the silver film on the polymer surface were studied after sintering using the scanning electron microscope (SEM) Prisma E (Thermo Scientific, Brno, Czech Republic) in high vacuum mode (~5 × 10^−4^ Pa) with an accelerating voltage of 3.5 kV. The sample was frozen in liquid nitrogen for 20 min; a cut was then made with a sharp scalpel from the side opposite to the silver layer. The sample was fixed on an L-shaped holder using carbon tape, so the resulting slice was perpendicular to the optical axis of the microscope.

The X-ray diffraction of the ink sample powder was studied after thermal (t = 60 °C) treatment of the ink portion under vacuum for one hour. X-ray diffraction data was collected using a Rigaku Smartlab SE diffractometer (Rigaku, Tokyo, Japan) equipped Cu Kα radiation (1.5406 Å). A Bragg angle 2θ ranging from 20° to 80° with a 0.02° step. 

Electronic spectra of the ink were recorded on a Varian Cary 50 Scan spectrophotometer.

The hydrodynamic diameter of nanoscale silver particles was determined by dynamic laser light scattering (DLS) using ZetaSizer Nano (ZEN 3600) equipment (Malvern Instrument, Worcestershire, UK) supplied with a 4 mW He–Ne laser (λ_0_ = 633 nm). Measurements were performed at 20 °C and a fixed scattering angle of 173^0^. Before measurement, the samples were diluted five times with aqueous ammonia and thoroughly de-dusted by filtration through “Millipore” membrane filters with an average pore diameter of 8 microns. Each sample was measured in 10 runs. In accordance with the software, the runs that contained the poorest data were automatically rejected while the remaining runs were analyzed and used in the final measurement calculation. The average value was determined for each sample; the standard deviation during measurements did not exceed 6%.

### 2.4. SAXS Studies of Colloid Particles

Small-angle X-ray scattering (SAXS) of the ink was studied using Rigaku SmartLab SE. The scattered X-ray intensity is represented as a function of the scattering vector *q* = 4*π* (*θ*)/*λ*, where 2θ is the angle between the wave vector of incident and scattered radiation, and λ is the wavelength. The radiation source is a ceramic X-ray tube with a copper anode, with a power of 2.2 kW (Cu Ka1 + Ka2). The scattered radiation was detected by a one-dimensional position-sensitive detector with a dynamic range of over 108 pulses/s. The sample–detector distance was approximately 320 mm. The observed angular range was 0.05–0.7 Å^−1^. The scattered radiation of a quartz capillary filled with ammonia was considered as a background and was subtracted from the scattering curve by a solution of silver particles during data processing. The ink solution was studied after 7 days of storage at t = 3 ÷ 5 °C; the SAXS experiment was conducted using a dilute solution of silver particles with a mass concentration approximately equal to 1%.

### 2.5. Theoretical Model of SAXS: Model of Aggregated Polydisperse Spheres (APSs)

Silver in the form of spherical particles with ranged sizes (polydisperse system) was considered. The intensity of scattered radiation can be calculated using the following [66]:

(1) $$I\left(q\right)=S_{c}\int_{0}^{\infty }V^{2}\left(R\right)P^{sph}\left(q,R\right)D\left(R,\sigma \right)dR+B$$

Here, S_c_ is the normalization factor, V(R) is the volume of the sphere with radius R and B is the constant of the background scattering. The form-factor of spherical particles with radius R can be calculated as follows:

(2) $$P^{sph}\left(q,R\right)={\left[3\frac{sin\left(qR\right)-qRcos(qR)}{{\left(qR\right)}^{3}}\right]}^{2}$$

We chose the Schultz–Zimm function to describe the spherical particle size distribution, since it is possible to describe asymmetric distributions with it by using the following formula:

(3) $$D\left(R,z\right)={\left(\frac{R}{<R>}\right)}^{z}\frac{{\left(1+z\right)}^{\left(1+z\right)}}{\Gamma (1+z)}e^{-\frac{(1+z)R}{<R>}}$$

Here, <R> is the mean value of the sphere radius, and the root-mean-square deviation is σ = (1 + z)^−1/2^ <R>^2^, where Γ(1 + z) is the gamma function.

## 3. Results and Discussion

The silver ink synthesis is based on the modified Tollens technique described in work [50]. The idea of the technique is to obtain a stable solution with precursors of metallic silver in the form of complexes with ligands that can be easily evaporated from the surface by slightly increasing the temperature.

Below are the main chemical reactions in precursor formation:

2 AgCH_3_CO_2_ + 2 NH_4_OH → Ag_2_O + 2 NH_4_CH_3_CO_2_ + H_2_O

Ag_2_O + 4 NH_3_ + 2 NH_4_CH_3_CO_2_ + H_2_O → Ag(NH_3_)_2_CH_3_CO_2_ + 2 NH_4_OH

Silver oxide is formed intermediately in the reaction of silver acetate with aqueous ammonia. When there is excess ammonia, silver in the oxide form passes into a soluble complex. The reduction of non-complexed silver ions is carried out by introducing a small amount of formic acid into the aqueous solution. The solution color changes from transparent to gray due to the formation of metallic silver, which slowly precipitates. The printing ink after the removal of the silver precipitate does not contain silver particles and must be a clear solution. During the printing process, as the volatile ligands evaporate due to moderate heating (above 50 °C), silver ions are reduced to atomic Ag in the presence of ammonium formate:

2 Ag(NH_3_)_2_CH_3_CO_2_ + NH_4_HCO_2_ → 2Ag + 5NH_3_ + 2CH_3_CO_2_H + CO_2_ + H_2_O

**Figure 1:**
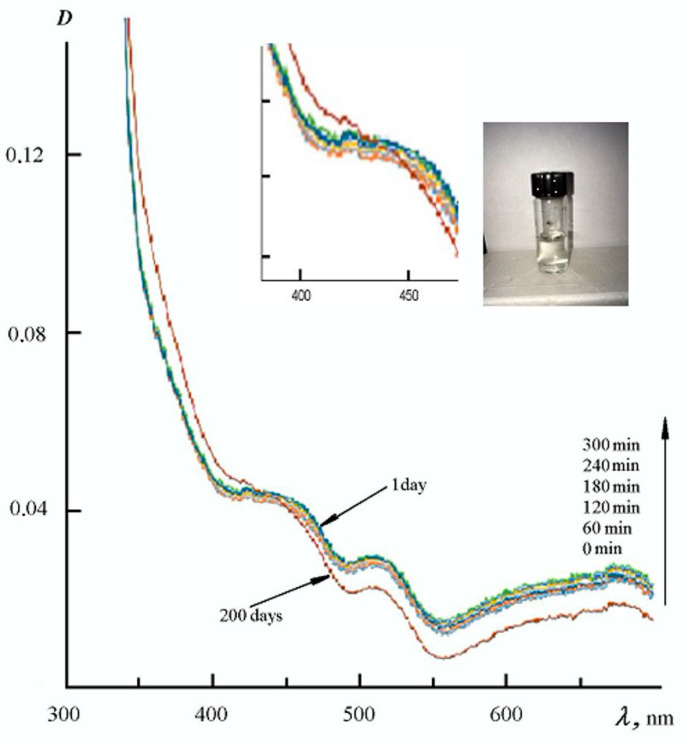
UV-VIS spectra for silver-based ink (photo) over time at temperatures of 20 °C, time 0 min–300 min—1 day; 5 °C, time from 1 to 200 days.

During the synthesis of ink, all conditions like cooling, intensive mixing, dropwise addition of reagents and strict adherence to their ratio were controlled, since violation of these conditions led to the formation of a significant silver precipitate. In our experiments, the obtained ink became transparent after two hours of preparation and filtration and, as a result, looks like a clear solution. However, their electronic spectrum contains absorption peaks in the visible light region (Figure 1), belonging to colloidal silver dispersions. The obtained spectrum was interpreted in terms of the surface plasmon resonance (SPR) theory for nanoscale metallic silver [67,68]. Thus, according to [67], the strong SPR absorption peak at ~430 nm is attributed to spherical Ag nanoparticles. Peaks in the λ > 500 nm region may indicate both the presence of relatively large spherical nanoparticles (50–100 nm) [69] and the presence of various crystalline forms of nanosilver. For example, in [70], hexagonal silver nanoplates were obtained and studied, the solution spectrum of which has a characteristic peak at 586 nm. At the same time, it is known [68] that the SPR bands can be strongly shifted up to the near-IR region when the aspect ratio of the nanoplates reaches a certain value.

Thus, the ink’s electronic spectrum shows that during the synthesis, part of the reduced silver does not participate in a complexation reaction, but forms colloidal nanoparticles, which probably have different crystalline shapes and sizes. However, the optical density value in Figure 1 shows that colloidal nanoparticles occur to an insignificant extent. Figure 1 also demonstrates the high chemical stability of the obtained ink: the spectrum is slightly transformed within one day at ambient temperature (20 °C). During storage for 200 days at T = 3 ÷ 5, small changes were also observed, including a drop in the total optical density of the solution in the region of 430–700 nm. This change is apparently caused by a decrease in light scattering due to the weak aggregation and sedimentation of particles. In addition, an insignificant change in the ratio of equilibrium crystalline forms of nanosilver was observed.

The size of silver particles is an important technical characteristic of the ink; therefore, the obtained solutions were studied by the SAXS and DLS methods. According to the DLS data, the particle size distribution in the solution a few hours after its preparation has a bimodal character with an approximately equal content of fractions with a hydrodynamic radius of ~4 nm (particle fraction ~53%) and ~26 nm (~47%).

**Figure 2:**
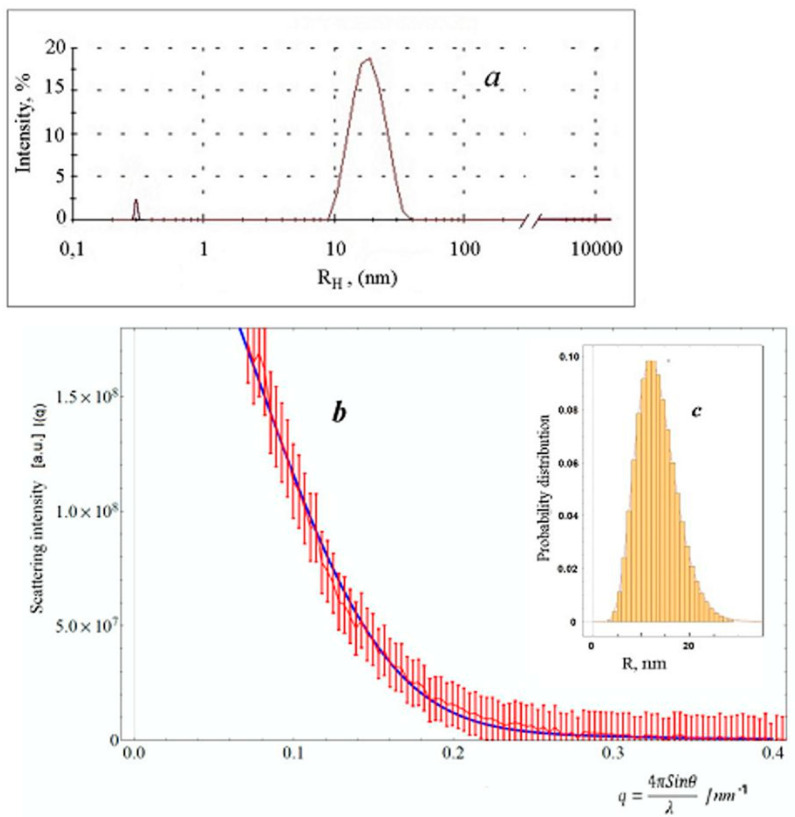
(**a**) Particle size distribution of silver ink particles, according to the DLS data. The concentration of Ag is 2.6 wt.%. (**b**) The scattering curve of a silver ink sample with an error bar (red), and the theoretical intensity curve calculated according to Formula (1) (blue). (**c**) The histogram and distribution function (*R*,) of silver particles (SAXS). The concentration of Ag is 1.0 wt.%.

Eventually, the particle size distribution transforms into the single peak distribution. Thus, after 7 days of storage, the main fraction contains approximately 95% of all particles with a mean radius value equal to 18.7 ± 2.5 nm (Figure 2c). Further, while maintaining the unimodal distribution, a tendency to aggregation is observed over time: the average particle size increases and the polydispersity of the system improves slightly, which follows the broadening of the scattering intensity peak. After 15 days, the average hydrodynamic radius of nanoparticles reaches ~40 nm and maintains this value up to 170 days, which indicates sufficient stability of the resulting silver ink when stored at low temperatures (3 ÷ 5 °C).

The SAXS experiment yields approximately the same result in terms of particle size distribution in the silver ink. The experimental data approximation calculated using Formula (1) is shown in Figure 2a. The proximity of the experimental and calculated curves indicates the adequacy of the chosen model. The particle size distribution function (*R*) (see Formula (2)) is shown in Figure 2b. The calculated mean value of particle radius <R> = 13.32 nm and its root-mean-square deviation σ = 4.23 nm. Although there are some discrepancies between the size data obtained by the DLS and SAXS methods, these could be explained by the presence of silver crystalline structures of much larger sizes (Figure 1).

**Figure 3:**
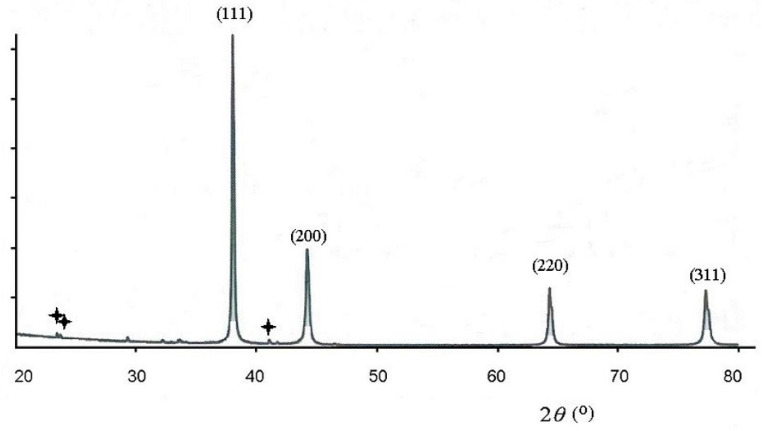
X-ray diffraction patterns from reactive silver ink annealed at 60 °C for one hour in vacuum.

Phase content of the reduced silver in the ink was investigated by X-ray diffraction analysis (XRD), and the diffraction pattern is shown in Figure 3. Its analysis shows that there is only one reduced product, consisting of metallic silver with a face-centered crystal lattice. The diffraction peaks located on 2*θ* equal to 38.2°, 44.4°, 64.4° and 77.5° are related to hkl indexes (111), (200), (220) and (311), respectively. Comparison of these peaks to the JCPDC database reveals that the investigated sample consists of silver crystals (bulk silver phase) (JCPDC no. 04-0738). A negligible amount of the initial silver acetate was indicated (reflexes are marked with an asterisk), which shows the completeness of the silver reduction reaction at a relatively low temperature (60 °C). No significant diffraction peaks are detected from any impurities, especially not from the Ag_2_O oxide. The crystal lattice period *a* calculated from the peak (111) of the diffraction pattern is equal to *a* = 0.40801 nm, which practically coincides with the table value (JCPDC, *a* = 0.40862). The grain size of the silver crystals was estimated from the broadening of reflections in Figure 3 using the Scherrer formula:

*d* = 0.89 *λ*/*B* cos *θ* (4)

where *d* is the particle size, *λ* is the X-ray wavelength, *θ* is the Bragg angle and *B* corresponds to the width of the full peak at half maximum (FWHM). The calculated size of silver crystals corresponds to a value of 44.72 nm. This value is close to those obtained by the DLS and SAXS methods. Thus, it can be concluded that the reduced agglomeration of silver leads to a highly monodisperse system both in the solution and after its removal.

Therefore, the solution stability, the small silver particle size (r ≤ 50 nm) and their low concentration (see Figure 1) provide acceptable ink quality for inkjet printing. It should be noted, however, that one of the disadvantages of this ink is the difficulty in conservation, which requires darkness and low temperatures. Inkjet printing, as is known, demands several requirements relating to the physical characteristics of the ink. In our research, we measured the following solution properties: viscosity (3 cP) and surface tension (1.74 mN m^−1^). We also measured the pH of 10.66 and silver content (13.9%) for the ink solution and also determined that the ink stability range pH = 10 ÷ 13. The ink synthesized by us has a rather high concentration of silver and a viscosity 4–7 times lower than, for example, the viscosity of the silver dispersions studied in [39,71]. However, compared to the reactive ink described in [60] and the classic study [50], the viscosity of our system is twice as high and almost an order of magnitude higher, respectively. At the same time, the solution obtained by us has very low surface tension, and this parameter is an order of magnitude higher than in other described samples [39,60,71]. Therefore, the main physical characteristics of the silver ink totally satisfy the requirements of inkjet printing [39,60,71,72].

## 4. Inkjet Printing and Heat Treatment

When choosing a printer model, we took into account viscosity and pH to avoid ink splashes in the selected printing conditions. It was found that to produce high-quality printed lines, the surface of the polyimide film requires essential treatment to increase hydrophilicity. Various chemical reagents were used for this purpose, but etching with a sodium hydroxide solution turned out to be simpler and more acceptable. Sodium hydroxide chemically modifies the surface, probably through a polyimide ring opening reaction. Indeed, as a result of treatment, the adhesion of the ink is increased sufficiently for high-quality printing. This was also expressed in a decrease in the contact wetting angle from 49.6° for the original surface to 43° for the modified one. These data guide us towards the development of methods to improve the contact properties of the substrate surface. Our immediate plans include the use of laser ablation and plasma treatment. We also intend to expand the range of polymer substrates for printing.

**Figure 4:**
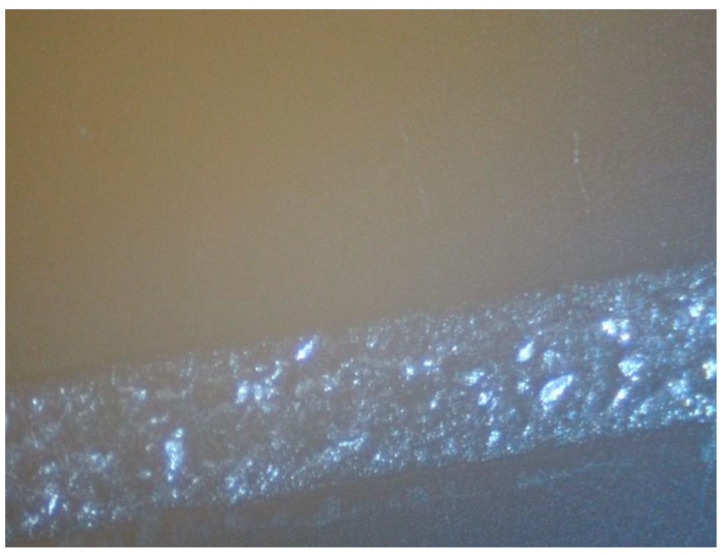
An optical microscope image of the cured ink surface magnified 6 times.

The production of electrical circuits requires multi-pass printing to obtain continuous lines. Patterns formed on the printer in three passes have an electrical resistance five times lower than those obtained in one pass (sintering temperature is 150 °C). Further printing was conducted in three passes; the width of the resulting track was 5 mm. The images obtained with silver ink on a 3D printer were subjected to sintering, which was performed in two stages. Initially, the ink was kept at 60 °C until dry, then the temperature was increased to the required value, determined by the substrate material. Increasing the sintering temperature to 210 °C lowers the resistivity several times. Excessive heating in the first sintering stage with a temperature above 100 °C can lead to the appearance of bubbles in the cured ink (Figure 4), which is reflected in the electrical conductivity of the patterns. The surface resistance after sintering at 210 °C without pre-baking was 10.3 Ω/sq, while that with pre-baking was 3.48 Ω/sq.

**Figure 5:**
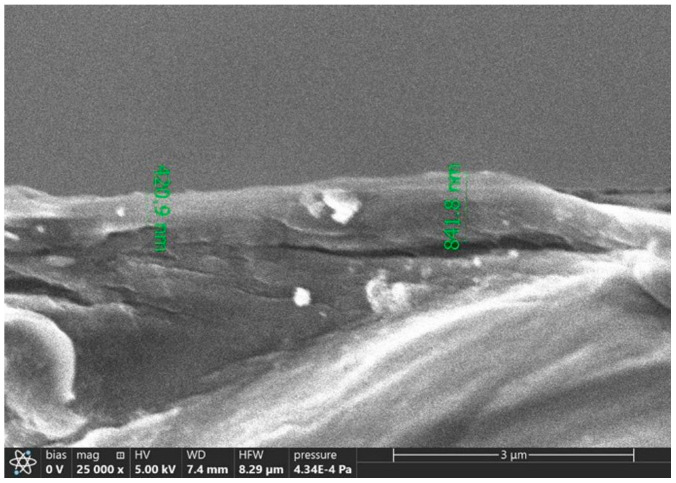
SEM images of the sintered ink cross section on a polyimide film. Two-stage sintering at temperatures of 60° and 150 °C.

During sintering up to 100 °C, a glossy silver layer (silver mirror) is formed. The surface does not change, remaining glossy and without bubbles or other defects after the second sintering stage at a higher temperature. A very dense silver layer is formed without micro-globules larger than 0.2 μm (Figure 5).

The morphology of the sintered layer in most of the studied inks from other manufacturers results in a rather non-compact layer containing numerous cavities and well-defined grains of 1–2 microns. It should be noted that this feature is also typical of reactive inks [52,60,63,73]. The authors note a significant change in morphology with an increase in sintering temperature, which is expressed in a decrease in porosity and in closer contact and coalescence of particles. As the temperature increases, the formation of silver agglomerates and clusters combined in interpenetrating networks is observed. Extended channels for electron transport appear, which reduce the electrical resistance of the material. At the limit, when the temperature rises above 200 °C, a bulk metal structure is formed. Formation of a continuous silver layer naturally leads to a sharp increase in electrical conductivity.

The samples we obtained demonstrate continuous structure of the bulk, even at relatively low annealing temperatures. The unusual morphology of the sintered patterns could be due to both the presence of nanoparticles in the initial ink and the stage-by-stage annealing temperature regime. Nanoparticles could act as seeds for the crystallization of metallic silver on the substrate surface. The low processing temperature reduces the evaporation rate of the solvent and promotes the growth of silver crystal domains. Thanks to the low drying and sintering temperature, the ink can be used to create metal conductive elements on flexible polymer films.

Measurements of the surface resistance of printed lines were carried out for samples obtained at different sintering temperatures (Table 1). From these data, it appears that the resistivity of the printed structures decreases sharply in the annealing temperature range of 100–120 °C, and changes insignificantly with a further increase in temperature. That is, the operating temperature range for the synthesized ink can be 120 ÷ 150 °C, which makes it possible to use a wide variety of substrates for printing.

The resistivity values (Table 1) after annealing at temperatures ≥120 °C are equal in order of magnitude to the data described in work [46] on the conductivity of the best reactive silver ink.

## 5. Conclusions

In the present paper, we introduce the production method of reactive silver ink for inkjet printing (Voltera V-One 3D printer) on a flexible polyimide film. Water-based ink with 13.9% silver mass concentration was obtained. Due to the collateral process of reduction of silver in the solution, Ag nanoparticles with a size of ~50 nm or smaller are formed, which, however, do not interfere with the printing process. The main advantages of the developed reactive ink are the sufficiently low sintering temperature and the formation of a dense, continuous layer of silver in the micrometer range. This feature determines the small values of the conductive line resistivity and shows the potential effectiveness of the ink application achieved for inkjet printing using a wide range of polymer substrates.

## Figures and Tables

**Table 1:** The dependence of the surface resistance ρ_sq_ on the sintering temperature.

T, °C	ρ_sq_, Ω/sq
100	8.9
120	3.54
150	3.91
180	3.55

## Data Availability

Data are contained within the article.
